# Optimizing ventilator support in severe bronchopulmonary dysplasia in the absence of conclusive evidence

**DOI:** 10.3389/fped.2022.1022743

**Published:** 2022-11-24

**Authors:** Audrey N. Miller, Matthew J. Kielt, George T. El-Ferzli, Leif D. Nelin, Edward G. Shepherd

**Affiliations:** ^1^ Division of Neonatology, Department of Pediatrics, Ohio State University, Nationwide Children’s Hospital, Columbus, OH, United States; ^2^ Comprehensive Center for Bronchopulmonary Dysplasia, Nationwide Children’s Hospital, Columbus, OH, United States

**Keywords:** bronchopulmonary dysplasia (BPD), ventilator, physiology, outcomes, severe, chronic

## Introduction

Bronchopulmonary dysplasia (BPD) remains the most common late morbidity of infants born preterm (1–3). BPD occurs along a spectrum of disease severity, and we will refer to severe BPD as the need for invasive mechanical ventilation at 36 weeks post-menstrual age (PMA), which in a contemporary definition of BPD is classified as grade 3 BPD (1). Infants with severe BPD are at higher risk for mortality, significant morbidities, neurodevelopmental impairment, total ventilator days, medication usage, and rates of procedures (tracheostomy and gastrostomy tube) compared to infants with less severe forms of BPD (1, 2, 4–6).

**Figure 1:**
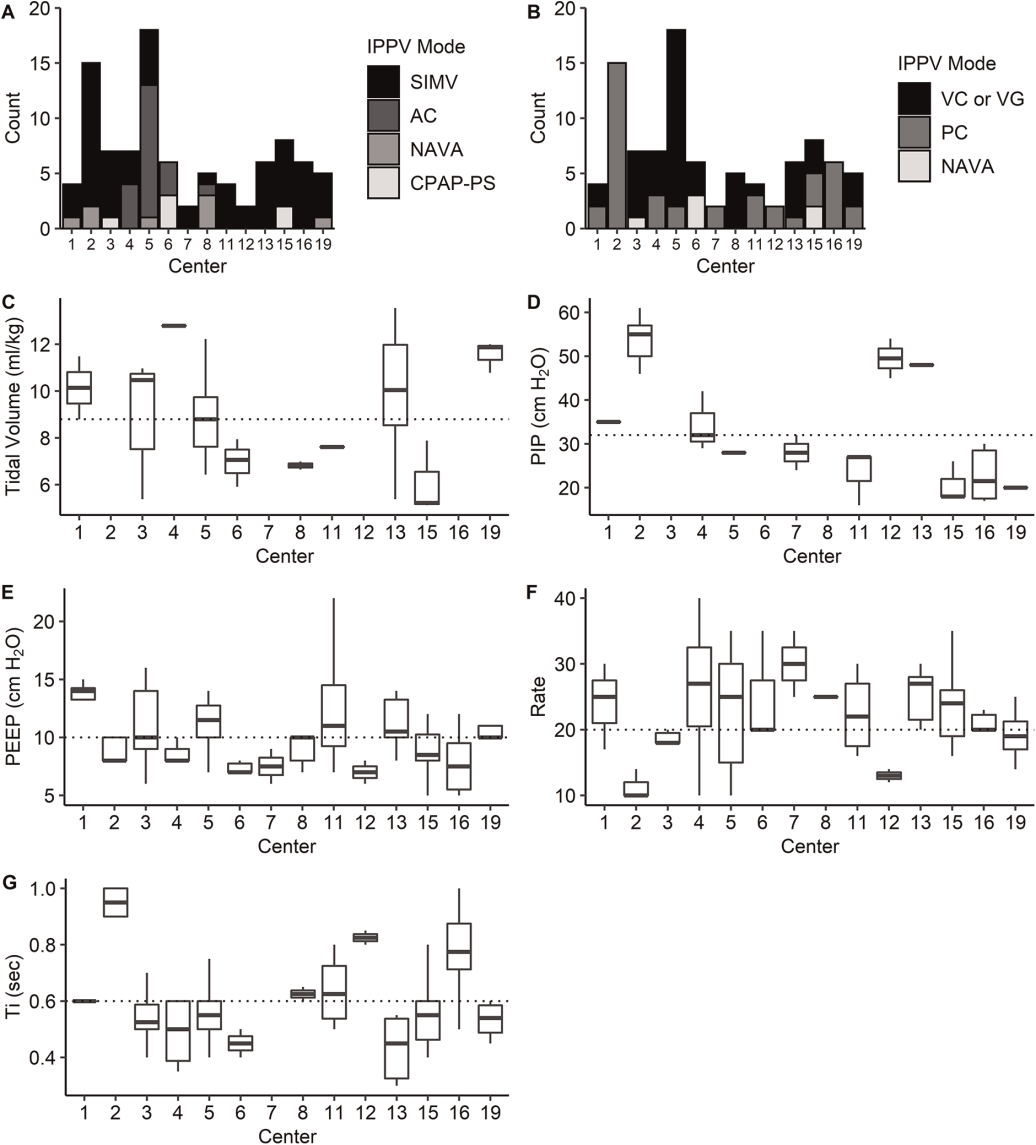
The distribution of IPPV modes and settings stratified by center. (**A**) Mode selection varied significantly by center when comparing the use of AC, SIMV, NAVA, and CPAP-PS (*P* < 0.0001, *χ*^2^ test). (**B**) Mode selection varied by center when comparing VC/VG, PC, and NAVA modes (*P* < 0.0001, *χ*^2^ test). (**C,D**) Among centers, V_T_ (ml/kg) did not differ significantly for patients on VC/VG ventilation (*n* = 49; *P* = 0.09) (**C**); however, PIP differed significantly for patients on PC ventilation (*n* = 40; *P* = 0.002) (**D**). (**E,F**), PEEP differed significantly for subjects receiving IPPV (*n* = 91; *P* = 0.001 (**E**), as did rate (*n* = 83; *P* = 0.001) (**F**). (**G**) Ti differed significantly for patients receiving time-cycled ventilation (*n* = 83; *P* < 0.0001). The median for study population is represented by the dotted line for each setting. PS, pressure support. Reprinted from McKinney et al. (9).

Given the lack of high-level evidence regarding the optimal management of infants with established severe BPD, care is highly variable across centers and regions (7). It is acknowledged that ventilator approach, settings, and weaning methods for the infant with established severe BPD vary widely between both providers and centers (6, 8). There remain no prospective, randomized controlled trials that support widespread application of any given respiratory approach. One multi-center point prevalence study evaluated ventilator modes and settings in infants with severe BPD, and as expected significant variation in ventilation setting and mode selection was reported (9). This variation was noted among sites in the BPD Collaborative, a collective of hospitals with interdisciplinary BPD programs, and is illustrated in Figure 1 (9).

Furthermore, it has been reported that there is also large variation in the rates of death or tracheostomy in infants with severe BPD. Murthy et al. (7) found in a large, multi-center cohort of infants with severe BPD, the center itself was an independent risk factor for death or tracheostomy, suggesting marked practice variation among clinicians, particularly with regards to tracheostomy. There is currently no uniformly accepted guidance regarding tracheostomy indications or timing, or more importantly evidence on how tracheostomy, or timing of tracheostomy, influence respiratory and neurodevelopmental outcomes (7, 10).

In the absence of high-quality evidence, therapeutic and ventilation strategies should be based on the information available related to respiratory physiology and minimizing complications.

## Available information

### Natural history of disease

BPD is a chronic disease that develops and then evolves over time. The precise pathogenic mechanisms that lead to the development of BPD, and the disease course, remain uncertain. There are several risk factors that predispose infants to the development of BPD, including lower gestational age at birth, sepsis, surgical necrotizing enterocolitis, and the need for mechanical ventilation (11). The pathophysiology of BPD is influenced by lung development, lung injury, and repair mechanisms that together ultimately lead to a heterogeneous disease with significant variability across the lung (12). This heterogeneous lung disease includes alveolar simplification, macrocystic disease, hyperinflation, microcystic disease, fibrosis, pulmonary vascular disease, and/or atelectasis (12). Infants with severe BPD can have significant parenchymal lung disease, large airway disease, and/or pulmonary hypertension, often with more than one of these disease components (13).

### Respiratory physiology

The respiratory physiology observed in severe BPD is markedly different than that seen in the preterm infant with respiratory distress syndrome, which is a homogeneous lung disease. Infants with severe BPD as described above have a heterogeneous lung disease with areas of very different lung physiology (14). There are regions of the lung in severe BPD with a relatively normal compliance and resistance, and therefore a relatively normal (or in comparison to other lung regions a relatively short) time-constant (time constant = resistance × compliance). These regions of the lung are often in the minority in patients with severe BPD (15). The majority of the lung in these patients is characterized by regions with relatively high airway resistance and normal or high compliance, resulting in very long time-constants. These areas of lung are characterized by hyperinflation, airway obstruction, air trapping, and ventilation perfusion (V/Q) mismatch (16–18). The majority of infants with severe BPD demonstrate this element of obstruction, with 91 percent of infants with severe BPD in one cohort demonstrating evidence of airflow obstruction on pulmonary function testing (14).

The respiratory mechanics of severe BPD suggest that the ventilation strategy used acutely in preterm infants aimed towards prevention of BPD, which is characterized by small tidal volumes (Vt), fast rates, and short inspiratory times (Ti) will no longer be effective for patients with severe BPD. This “lung protective” strategy preferentially ventilates the relatively healthy lung regions, which make up the minority of cross-sectional area available for gas exchange. This happens at the expense of the diseased lung regions, which make up the majority of cross-sectional area available for gas exchange. Infants with severe BPD ventilated with this “lung protective” strategy therefore usually manifest signs of being under supported including increased work of breathing, hypoxemia, desaturation events or “spells”, and/or severe V/Q mismatch as manifest by a requirement for a very high fraction of inspired oxygen (FiO_2_) (11).

A “chronic” ventilator approach using a relatively slow-rate, high Vt, and long Ti, improves ventilation of the diseased lung regions, which make up the majority of the available gas exchange area and are characterized by a very slow time constant. By improving ventilation to the diseased regions of the lung, V/Q matching is improved, which is evidenced by the ability to wean the FiO_2_. Furthermore, the slow rate allows for a prolonged expiratory time to facilitate lung emptying and improve hyperinflation, which will not only increase Vt but also result in further improvement in V/Q matching (16). This strategy allows for improved gas exchange, reduced atelectasis, and decreased dead space ventilation in the majority of patients with severe BPD (11). While this strategy is not based on high-quality evidence, it is guided by the respiratory physiology seen in severe BPD, and is supported by clinical experience (15–18).

### Complications

Infants with severe BPD are at risk of in-hospital mortality (6). Pulmonary hypertension (BPD-PH) is seen in up to 25% of patients with severe BPD and is associated with increased risk for mortality (19). The risk of death after discharge in patients with severe BPD is likely highest in patients with tracheostomies and a history of BPD-PH (20, 21). Infection is also an important cause of both in-hospital and post-discharge mortality in this population (9).

Infants with severe BPD often have significant co-morbidities. These infants are at risk for growth failure, which is likely multifactorial and secondary to the level of nutritional support, medical management practices that suppress growth, chronic stress, and/or inflammation (22). Infants with severe BPD often require supplemental oxygen after discharge (23). Infants with BPD are at higher risk for re-hospitalizations compared to patients without BPD, often secondary to respiratory illnesses, with one study demonstrating 49% of infants with BPD required re-hospitalization in the first year of life (24). Infants with severe BPD are also at risk for developing blindness and hearing loss (25).

BPD is a known risk factor for long-term neurodevelopmental impairment (26). BPD has been independently associated with cerebral palsy in infants that require mechanical ventilation at 36 weeks PMA compared to infants with BPD not requiring mechanical ventilation at that time (27). Additional studies have shown that BPD patients are more likely to have microcephaly, behavioral, motor, and postural disturbances compared to controls (28).

Infants with severe BPD are also often subjected to treatment practices and therapeutics that have known risks for neurodevelopmental impairments. The use of analgesics and sedatives may have a detrimental effect on the developing brain as suggested by animal and clinical studies (29). Opioids may induce apoptosis in human microglial cells and neurons, and lead to long-term changes in brain function and memory (29). Exposure to midazolam has been shown to affect hippocampal development and long-term learning memory in survivors of neonatal illness (29).

In infants, touch is an important part of sensory-cognitive development, and the response to light touch is positively impacted by the number of supportive experiences and negatively impacted by the number of painful experiences (30). Preterm infants with a higher number of skin-breaking procedures (i.e., lab draws) from birth to term had lower cognitive and motor development at 8 and 18 months corrected age (31). This implies that repetitive pain-related stress and procedures are associated with worse neurodevelopment in the first 2 years of life (31). This evidence suggests that routine blood gas sampling may be associated with long-term harm in patients with severe BPD.

A respiratory support strategy that does not allow for patient comfort or ease of breathing leads to an inability to engage with the environment and make developmental progress. A chronic care model that focuses on adequate respiratory support with positive touch experiences, avoidance of noxious stimuli, early intervention programs, and a reduction in medications is key in positively impacting neurodevelopment in these high-risk infants. One study demonstrated that 56% of infants in a cohort of patients with moderate to severe BPD had no neurodevelopmental impairment using this type of chronic care model (32).

## Acute to chronic care transition

Patients with severe BPD are often cared for in acute care settings (11). Within the practice of acute critical care, the fundamental goal is often to wean respiratory support based on the rationale that a shorter duration of support is associated with a shorter length of stay, fewer complications, and better overall care. In this acute care setting, the goal for patients with severe BPD is often to decrease ventilator settings and extubate to non-invasive support as soon as possible. Additionally, escalation of support may be viewed as a failure of care. For very preterm infants early on in life, this acute care approach and lung protective ventilation strategy aimed towards prevention of BPD are appropriate (18). However, those infants who cannot be weaned from invasive respiratory support will require chronic ventilation. Continued efforts using an acute care approach of trying to wean support in these infants may result in atelectasis, worsening gas exchange, escalating FiO_2_ requirements, and increased work of breathing.

Additionally, an assumption in critical care is that we can use discrete and objective data to reassure providers that current care goals are being met, or redirect providers to change the care plan. This leads to the thought process that more data is better (blood gases, lab monitoring, imaging), and often leads to more acute clinical changes. These data are helpful in the acute setting where the goal is BPD prevention. However, in patients with established severe BPD, respiratory stability may be better reflected in clinical variables that can be measured non-invasively at the bedside, such as FiO_2_ needs, work of breathing, growth, parental reports of comfort, and ability to tolerate cares and developmental therapies.

There is no definitive evidence upon which to base the decision of optimal timing to transition from acute models of respiratory support to chronic phase ventilation (CPV). However, there is evidence that resistance increases in chronically ventilated premature infants (16). Therefore, we believe that transition to CPV should occur when clinical indicators suggest that premature infants are no longer responding well to standard lung protective ventilator strategies. This is frequently described as clinical instability, worsening hypoxia despite increased mean airway pressure, and frequent desaturations or “spells.” In addition, other signs of increased resistance include hyperinflation on chest radiography, positive response to bronchodilators and/or corticosteroids, and worsening status on “gentle ventilation” modes like HFOV, or high-rate, low tidal volume ventilation. This may occur relatively early in the disease progression (near the first month of life), or it may occur as late as 32–36 weeks PMA.

Thus, infants with severe BPD require transition from an acute care model to a chronic care model. Provision of adequate respiratory support without rapid weaning becomes critical, and allows for adequate gas exchange, reduced work of breathing, and improved overall growth and development.

## Principles of respiratory care of infants with severe BPD

Given that there is no conclusive evidence for how to manage infants with severe BPD we have based our approach on the best available information relating to the respiratory physiology and expected natural history of the disease process.

### Ventilator management

The best available evidence suggests that severe BPD is a heterogeneous lung disease that can be adequately described using two functionally distinct compartments. The first lung compartment is relatively healthy with normal compliance and resistance. The second, and predominant, compartment is damaged and has high resistance with relatively normal compliance. As a result, the time constant for the second or “slow” compartment is very long leading to prolonged exhalation and air-trapping. Because of this heterogeneity, ventilator management is difficult and does not follow the typical algorithms used in the “lung protective strategies” discussed above.

There is wide provider variability regarding ventilator mode selection for patients with BPD, even among centers in the BPD Collaborative (9). Our preference is a pressure synchronized intermittent mandatory ventilation (SIMV) with pressure support mode. We find that pressure control allows us to adequately ventilate in the setting of large air leaks around the endotracheal tube affecting reliable Vt delivery, which is common in this population. However, the underlying principles of CPV work regardless of which mode is selected, and would apply to centers using volume based ventilation.

The diseased lung compartment, with a slow time constant, is managed by allowing full exhalation with each set ventilator breath. Specifically, the ventilator rate must be set to allow five exhalatory time constants between breaths. In practical terms, this requires a ventilator rate of somewhere between 10 and 16 breaths per minute for infants with the most severe forms of BPD. Such rates allow 3–5 s of exhalation which in most cases will be adequate to achieve full exhalation. As a result, to achieve adequate minute ventilation, the tidal volume given with each ventilator breath must be high. Pressure settings to achieve adequate minute ventilation (tidal volume × rate) in this very ill population often are greater than 40 cm H_2_O.

For centers that utilize volume based ventilation, a target Vt within the range of 10–15 ml/kg is often needed, although this may be higher in infants with the most severe forms of disease. A good indication of adequate Vt in this population is good chest rise with ventilator breaths and comfortable work of breathing. Targeting these tidal volumes often leads to the need to set a higher peak pressure limit, as higher pressures (even exceeding 40 cm H_2_O) may be needed.

The appropriate level of positive end-expiratory pressure (PEEP) needed for patients with severe BPD remains to be determined, and a wide practice variation exists among centers (9). Even in the setting of hyperinflation, the set PEEP should be high (≥8 cm H_2_O) to maintain functional residual capacity, avoid atelectasis, and improve gas exchange (11). Some patients may require an even higher PEEP in the setting of tracheomalacia, bronchomalacia, or small airway malacia. Some patients may benefit from brief periods of higher PEEP to mitigate airway collapse, often in the setting of agitation or passing of stools (12). Intrinsic PEEP may contribute to patient/ventilator dyssynchrony, which may be improved by increasing the set PEEP (33).

Pressure support breaths where the patient sets their own Ti, additionally allows for ventilation of the healthy lung compartment. This combination of addressing the diseased and healthy lung compartments allows for more equal distribution of ventilation and thereby significantly improves V/Q matching. Please see Table 1 for typical CPV settings in this population.

**Table 1:** Example of chronic phase ventilation settings for the most severe forms of BPD.

Respiratory rate	10–16 breaths per minute
Inspiratory time	0.7–1.0 s
Peak inspiratory pressure	As needed, often 40–45 cm H_2_O or higher
Positive end-expiratory pressure	As needed, often 8–12 cm H_2_O or higher
Tidal volume	10–15 ml/kg or occasionally higher in the very worst forms of disease

### Ventilator weaning

Severe BPD is a disease with a protracted course which improves over time and successful weaning usually occurs with improvements in the rate of linear growth. Linear growth reflects overall nutritional status (34), is associated with successful respiratory support weaning (35), and is associated with improvement of lung function (36). Thus, weaning should be done slowly and cautiously. Our approach is to escalate ventilator support until the infant is comfortable, interactive, well saturated, and able to make developmental progress. Once we have achieved these settings, we focus on weaning oxygen until we achieve good length growth on a FiO_2_ of 0.4 or less. We do not believe that high ventilator peak inspiratory pressures (PIP) preclude an attempted extubation, and therefore do not routinely wean PIP prior to an extubation attempt. However if PIP weaning is attempted prior to extubation, a slow approach with changes only every 1–2 weeks is usually needed. On the other hand, we have found success with weaning the PEEP to 8 cm H_2_O prior to an attempted extubation to nCPAP. Our practice is to extubate to nCPAP at a level of 8 cm H_2_O, although we recommend extubating to the non-invasive device most successfully used in each center, which may include non-invasive positive pressure ventilation and/or high flow nasal cannula.

### Weaning from non-invasive support

Convalescence on non-invasive support typically occurs at about the same rate as improvement on the ventilator, and therefore prolonged periods of non-invasive support may be necessary. Our practice is to maintain nCPAP until we achieve steady linear growth and good developmental interactions on a FiO_2_ less than 0.3, at which point an attempt at low flow nasal cannula oxygen may be appropriate. We transition from nCPAP of 8 cm H_2_O directly to nasal cannula, without prior weaning of the nCPAP level. However, other centers may take a step wise approach to weaning nCPAP and/or change to high flow nasal cannula before reaching low flow nasal cannula. Regardless of the approach, the goal while weaning non-invasive support should again focus on continued developmental advancement and good linear growth.

### Respiratory assessment

While there is no evidence on the optimal way to assess chronic respiratory status, there is evidence that the quantity of skin breaking procedures done during neonatal hospitalization is directly correlated with worse neurodevelopmental outcomes. Given that there is also evidence that capillary blood gases may not be an accurate reflection of steady state respiratory status, we no longer believe that there is a positive risk:benefit ratio favoring obtaining routine capillary blood gases in infants with severe BPD. Therefore, we rely almost entirely on clinical assessments of overall respiratory status, including steady state oxygenation, serial physical exams, and repeated assessments by bedside staff.

### Timing of tracheostomy

A fraction of infants with severe BPD will require tracheostomy to maintain a stable airway. There is no consensus on the optimal timing for tracheostomy and long-term outcomes are uncertain. Tracheostomy and chronic home ventilation have significant risks and are resource intensive. Therefore, we believe that it is imperative to take a systematic approach with an emphasis on avoiding tracheostomy placement if possible. Consequently, we generally recommend tracheostomy placement after an infant reaches 50–52 weeks PMA and has failed repeated extubation attempts after 40 weeks PMA.

### Additional considerations

There are a number of additional controversies in the management of severe BPD, including the optimal use of diuretics, bronchodilators, inhaled steroids, systemic steroids, and the most effective means of providing enteral nutrition. While these are beyond the scope of this review, given the chronic nature of BPD it is critical to continue to use and create consistent guidelines in order to decrease unintended variability among providers and to facilitate learning and analysis (37, 38). Additionally, this described physiology and management approach is specific to infants with severe BPD, and may or may not apply to infants with other chronic respiratory disease, such as congenital diaphragmatic hernia, pulmonary hypoplasia, or other interstitial lung disease.

## Conclusion

The goal of chronic respiratory care in severe BPD is to provide an optimal platform of respiratory stability and growth to improve long-term pulmonary and neurodevelopmental outcomes. Factors that contribute to improving outcomes should be done aggressively, and factors that impair outcomes should be eliminated if possible. A lack of weaning respiratory support should not be viewed as failure, but as an opportunity to achieve success while promoting growth, neurodevelopment, and long-term outcomes.
